# Dynamic Illumination and Visual Enhancement of Surface Inspection Images of Turbid Underwater Concrete Structures

**DOI:** 10.3390/s25185767

**Published:** 2025-09-16

**Authors:** Xiaoyan Xu, Jie Yang, Lin Cheng, Chunhui Ma, Fei Tong, Mingzhe Gao, Xiangyu Cao

**Affiliations:** 1 State Key Laboratory of Water Engineering Ecology and Environment in Arid Area, Xi’an University of Technology, Xi’an 710048, China; 2 XAUAT Engineering Technology Co., Ltd., Xi’an 710055, China; 3 Nanjing Hydraulic Research Institute, Nanjing 210029, China

**Keywords:** underwater images, image detection, image enhancement, dynamic illumination

## Abstract

**Highlights:**

**What are the main findings?**

- The DIVE algorithm effectively addresses uneven illumination and color distortion in turbid underwater images by combining dynamic illumination correction and visual enhancement modules.
- The algorithm achieves real-time processing at 25 FPS for 1920 × 1080 resolution videos, making it suitable for embedded devices and underwater robotic inspections.

**What is the implication of the main finding?**

- DIVE provides a robust solution for underwater defect detection, significantly improving image quality in high-sediment environments (up to 500 g/m^3^).
- The method enhances feature extraction for concrete surface defects, such as cracks and holes, supporting applications in marine engineering and dam monitoring.

**Abstract:**

Aiming at the problem of image quality degradation caused by turbid water, non-uniform illumination, and scattering effect in the surface defect detection of underwater concrete structures, firstly, the concrete surface images under different shooting distances, different sediment concentrations, and different illumination conditions were collected through laboratory experiments to simulate the concrete surface images of a reservoir dam with higher sediment concentration and deeper water depth. On this basis, an underwater image enhancement algorithm named DIVE (Dynamic Illumination and Vision Enhancement) is proposed. DIVE solves the problems of luminance unevenness and color deviation in stages through the illumination–scattering decoupling processing framework, and combines efficient computing optimization to achieve real-time processing. The lighting correction of Gaussian distributions (dynamic illumination module) was processed in stages with suspended particle scattering correction (visual enhancement module), and the bright and dark areas were balanced and color offset was corrected by local gamma correction in Lab space and dynamic decision-making of G/B channel. Through thread pool parallelization, vectorization and other technologies, the real-time requirement can be achieved at the resolution of 1920 × 1080. Tests show that DIVE significantly improves image quality in water bodies with sediment concentration up to 500 g/m^3^, and is suitable for complex scenes such as reservoirs, oceans, and sediment tanks.

## 1. Introduction

Concrete structures that have been in service in turbid water environment for a long time, such as sea-crossing bridge piers and docks in marine environment, dams, flood gates, revetments and piers in river and lake environments, have been affected by adverse conditions such as erosion, chemical erosion, freezing and thawing for a long time, and their surfaces often have cracks and spalling problems, which pose a serious threat to the safe and stable operation of the structures [1]. Surface defects of concrete structures will not only weaken their durability but also cause more serious internal damage, thus threatening the overall safety of the structure [2]. However, the high risk of underwater inspection and the complexity of the water environment make the detection of underwater concrete surface defects extremely challenging [3,4].

Currently, robots equipped with image acquisition equipment and combined with machine vision technology for intelligent inspections have shown great potential in identifying and measuring surface defects of underwater concrete structures [5,6]. For example, Espinosa uses ROV (Remote Operated Vehicle) static underwater video and image collection platforms to collect underwater images [7]. Han et al. [8]. used underwater robots and image segmentation algorithms to automatically detect stilling pool damage.

**Figure 1:**
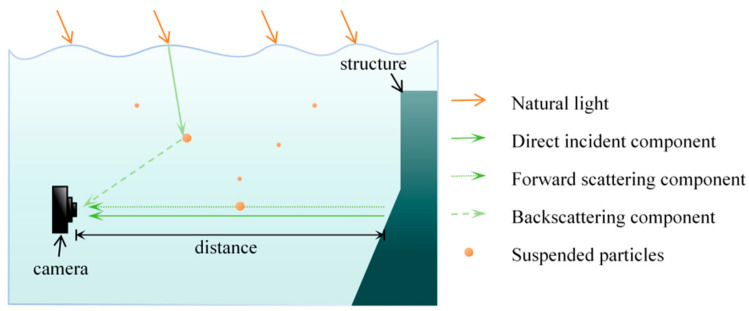
Schematic of underwater optical imaging.

However, underwater image detection faces physical challenges that are quite different from the atmospheric environment [9]. The schematic diagram of underwater optical imaging is shown in Figure 1. First of all, there are a large number of suspended impurity particles (such as plankton and sediment) and bubbles in the water body, which will cause strong scattering effects [10]. Scattered light forms a halo in front of the lens, causing non-uniform bright and dark areas of the image: bright areas lose details due to excessive light concentration, while dark areas mask features due to insufficient light. This phenomenon is called “underwater snow effect”, which is characterized by smooth transition between light and dark areas (such as Gaussian distribution), which makes it impossible for traditional sharpening algorithms to effectively restore details [11]. To make matters more complicated, different wavelengths of light attenuate differently in water (red light is the fastest, blue and green light are the slowest), resulting in color distortion and contrast reduction [12]. Quantitative analysis shows that unprocessed underwater images are significantly inferior to terrestrial images in structural similarity and other indicators [13]. Therefore, the development of a brightness correction algorithm adapted to the smooth transition characteristics of underwater light and dark becomes the key prerequisite for restoring image details.

At present, underwater image enhancement algorithms are mainly divided into three categories, namely non-physical model methods, physical model-based methods and deep learning-based methods. Among them, the non-physical model method is represented by Retinex theory and its improved model [14]. Retinex-like methods are based on illumination reflection theory and combined with nonlinear guided filtering to achieve illumination equalization. The key lies in adaptively estimating illumination components at different scales, which is prone to halo artifacts and loss of details in bright areas, such as Attenuated Color Channel Correction and Detail Preserved Contrast Enhancement (ACDC) [15], etc. The physical model-based approach introduces prior knowledge and uses the imaging model to inversely solve the degradation process [15], represented by the Dark Channel Prior (DCP) algorithm and its improved models, such as G-DCP. DCP can effectively suppress excessive enhancement by separating illumination components by counting the distribution of dark pixels in the image [16,17]. However, it is still difficult to avoid the failure of dark channel prior in high-scattering areas [18], and DCP-like methods have high computational complexity and are difficult to meet the requirements of real-time processing. Data-driven deep learning algorithms are gradually applied to computer vision tasks due to their deep network structure and good feature extraction capabilities [19]. However, due to the inability to obtain “truly clear” images in underwater environments, the performance based on deep learning methods always depends on training data quality, parameter adjustment, and learning framework [20], and the generalization ability and computational efficiency are limited. Currently, the primary research object in underwater image processing remains the main image data of shallow ocean areas [21,22]. There are few image monitoring datasets for reservoirs and dams with high sediment concentration and deeper water depth, and there are few targeted studies.

At the same time, application scenarios such as underwater robots and monitoring systems require low processing latency. However, existing algorithms face severe challenges; for example, the original DCP algorithm achieves only 2-3 FPS on embedded CPUs [23]. Deep learning models such as U-Net models require more than 500 ms at 1080 p resolution, which cannot meet real-time requirements [20]. Some studies improve computational efficiency by simplifying model parameters, such as LU^2^Net adopts axial depth separable convolution, which reduces parameters by 80% and reaches 12FPS on an i7-10750H CPU [24], but it is still difficult to achieve real-time processing using ordinary processors.

To sum up, existing underwater image enhancement methods face three contradictions: although Retinex-like methods have high computational efficiency, they have halo artifacts and detail loss in bright areas. Although DCP-like methods can suppress excessive enhancement, they are not robust due to dark channel prior failure, and deep learning models are limited by training data quality and computing resources. To solve these core problems, this paper develops the Dynamic Illumination and Vision Enhancement for Underwater Images (DIVE) algorithm. By constructing an illumination–scattering decoupling processing framework, DIVE processes Gaussian distribution illumination correction (dynamic illumination module) and suspended particle scattering correction (visual enhancement module) in stages. Through fast local gamma correction in Lab space and dynamic decision of G/B channel mean, the problems of non-uniform illumination and color cast are solved step by step. During the visual enhancement phase, the Contrast Limited Adaptive Histogram Equalization (CLAHE) parameters are dynamically adjusted through contrast and detail levels. For example, high-frequency discrimination blocks can be reduced by 50% to improve detail retention. To meet the needs of real-time monitoring, separate Gaussian convolution, Thread Pool Executor, and vectorized matrix operation technologies are utilized to achieve a processing speed of approximately 25 frames per second for 1920 × 1080 underwater detection video.

## 2. Dynamic Illumination and Vision Enhancement for Underwater Images

**Figure 2:**
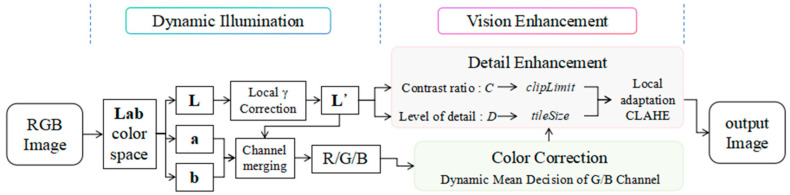
DIVE processing flow chart.

The DIVE algorithm is based on a lighting–scattering decoupling processing framework and comprises two core modules: dynamic illumination and visual enhancement. The dynamic illumination module, based on fast local gamma correction, employs Lab space downsampling and separated Gaussian convolution to address the issue of uneven underwater lighting effectively. The adaptive dual-channel visual enhancement module achieves color correction through dynamic decision-making of G/B channel means, and enhances details by combining the contrast-sensitive CLAHE algorithm. The processing flowchart is shown in Figure 2, and the detailed processing procedure is as follows.

### 2.1. Dynamic Illumination Based on Fast Local Gamma Correction

**Figure 3:**
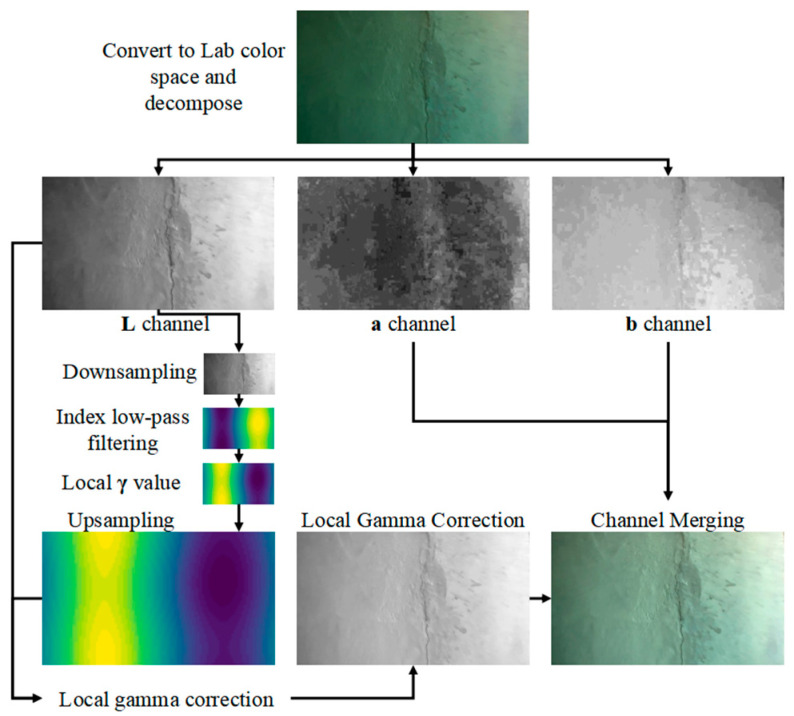
Dynamic illumination based on fast local gamma correction.

In order to solve the problem of uneven underwater illumination caused by natural light and auxiliary illumination, this paper proposes a dynamic illumination method based on fast local gamma correction. By performing low-pass filtering and local gamma correction on the L channel (brightness channel) in the original image Lab color space, the local nonlinear transformation based on illumination distribution is realized. The process is shown in Figure 3, and the specific steps are as follows.

The L channel in the Lab color space has the advantages of being independent of the chroma channels and possessing a larger dynamic range, making it more suitable for high dynamic range image enhancement tasks. Therefore, in the dynamic illumination stage, simply performing fast local gamma correction on the L channel itself can rapidly improve the brightness uniformity of the image.

(1) Lab color space conversion

Convert the image to the Lab color space to obtain the L (Lightness) channel of the image.

(2) L channel downsampling

To enhance computational efficiency and prevent information loss, multi-scale downsampling is applied to the L channel before low-frequency illumination estimation. This paper employs a multi-scale downsampling method based on the Gaussian pyramid, which better preserves the structural information of the image through layer-by-layer Gaussian smoothing and downsampling, avoiding artificial artifacts that may arise from simple interpolation.

Let the size of the original image’s **L** channel be (*h*,*w*), and define the target minimum side length as 64. Let the 0th layer be the original image, with **I**_0_ = **L**. The *k-th* layer image is obtained by performing Gaussian convolution and downsampling on the (*k −* 1)-th layer image:

(1) $$I_{k}=\mathrm{DOWN}\left(G_{\sigma }*I_{k-1}\right)$$

where $G_{\sigma }$ represents the Gaussian convolution kernel, and DOWN (·) denotes the downsampling operation.

Experimental results indicate that when the L channel of a 1080 p image is downsampled to 64 pixels using the Gaussian pyramid before subsequent processing, the total computation time is approximately 0.3% of that required for directly processing the original-sized image, representing a significant improvement in computational efficiency. Meanwhile, the multi-scale approach maintains better frequency domain characteristics, providing a reliable foundation for subsequent processing.

(3) Low-frequency illumination component estimation

The bright and dark regions in underwater images typically exhibit smooth transition characteristics (approximating a Gaussian distribution), making it difficult for traditional sharpening algorithms to effectively handle such gradual illumination variations [10].

This method applies Gaussian low-pass filtering in the downsampled low-resolution space to obtain a smooth brightness distribution. This approach not only effectively estimates the illumination distribution but also avoids edge blurring issues caused by using large-sized convolution kernels at the original resolution.

The standard deviation σ of Gaussian low-pass filtering directly determines the cutoff frequency. Through underwater image experiments under different working conditions, it is verified that for images with higher resolution (higher than 720 P), taking σ=2 can effectively filter out high-frequency noise (such as fine texture), while retaining medium- and low-frequency illumination components (such as shadow gradient).

To meet the real-time processing requirements, the Gaussian function is separated into two one-dimensional convolutions:

(2) $$G_{2D}(x,y)=G_{1D}(x)\cdot G_{1D}(y)=\left(\frac{1}{\sqrt{2\pi }\sigma }e^{-\frac{x^{2}}{2\sigma^{2}}}\right)\left(\frac{1}{\sqrt{2\pi }\sigma }e^{-\frac{y^{2}}{2\sigma^{2}}}\right)$$

Then $L_{\mathrm{filtered}\;}=L*G_{2D}=L*\left(G_{1D}⊗G_{1D}\right)=\left(L*G_{1D}\right)*G_{1D}^{T}$, where ⊗ denotes the outer product. The separate convolution is consistent with the results of the direct two-dimensional convolution.

(4) Calculate local gamma values and upsampling

According to the given $\gamma_{\mathrm{factor}}$ value (calibrated as 1.5 by various working condition images in this paper), the local gamma value $\gamma_{\mathrm{local}\;}(h^{\prime },w^{\prime })$ is calculated by using Equation (3):

(3) $$\gamma_{\mathrm{local}\;}(h^{\prime },w^{\prime })=1+\gamma_{\mathrm{factor}}\cdot \frac{255-L_{\mathrm{filtered}\;}(h^{\prime },w^{\prime })}{255-\mathrm{mean}\left(L_{\mathrm{filtered}\;}(h^{\prime },w^{\prime })\right)}$$

The resulting local gamma-value matrix is restored to the original size by bicubic interpolation upsampling to obtain $\gamma_{\mathrm{local}\;}(h,w)$.

(5) Gamma correction of the original **L** channel

Each pixel of the **L** channel is then gamma-corrected using the calculated local gamma values:

(4) $$L_{\mathrm{adjusted}\;}(h,w)=255\cdot {\left(\frac{L(h,w)}{255}\right)}^{\frac{1}{\gamma_{\mathrm{local}\;}(h,w)}}$$

Combine the adjusted **L** channel with the original a and b channels to obtain the brightness corrected image.

### 2.2. Adaptive Dual-Channel Visual Enhancement

#### 2.2.1. Color Correction Based on G/B Channel Mean Dynamic Decision-Making

Inspired by ACDC [15], the color cast is corrected by adjusting the proportional relationship of RGB channels to enhance the color contrast and dynamic range of the image. The specific steps are as follows:

Separate the red, green, and blue channels of the RGB image and normalize to [0, 1], adjusting the red (**R**) and blue (**B**) channels according to the green (**G**) and blue (**B**) channel mean.

If $\bar{G}>\bar{B}$, then

(5) $$\begin{array}{l} R\prime =R+(G-R)\cdot G\\ B\prime =B+(G-B)\cdot G \end{array}$$

Otherwise,

(6) $$\begin{array}{l} R\prime =R+(B-R)\cdot B\\ G\prime =B+(G-B)\cdot G \end{array}$$

R′, G′ and B′ represent adjusted channel values.

Calculate the sum for each channel:

(7) $$S_{R}=\sum_{x,y}R(x,y),S_{G}=\sum_{x,y}G(x,y),S_{B}=\sum_{x,y}B(x,y)$$

Calculate channel scale:

(8) $$\alpha_{R}=\frac{\max\left(S_{R},S_{G},S_{B}\right)}{S_{R}},\alpha_{G}=\frac{\max\left(S_{R},S_{G},S_{B}\right)}{S_{G}},\alpha_{B}=\frac{\max\left(S_{R},S_{G},S_{B}\right)}{S_{B}}$$

Scale the channel

(9) $$R_{\mathrm{scaled}\;}(x,y)=\min\left(255,\frac{R(x,y)\cdot \alpha_{R}}{255}\cdot 255\right)$$

Calculate $G_{\mathrm{scaled}\;}$ and $B_{\mathrm{scaled}\;}$ in the same way.

In order to meet the needs of real-time monitoring, Thread-Pool-Executor is used to implement parallel processing of RGB channels to improve processing efficiency. A matrix operation replaces the pixel-by-pixel operation, and the vectorization processing of color correction is realized, thus significantly reducing the computational overhead of pixel-by-pixel calculation.

#### 2.2.2. Detail Enhancement Based on Local Adaptive Mapping

In order to further enhance the details of underwater images, the contrast is enhanced by a local adaptive mapping function.

Firstly, according to the **L** channel after local gamma correction in dynamic illumination, the contrast and detail level feature values of the image are calculated:

First, the contrast ratio C of the image is calculated:

(10) $$C=\sqrt{\frac{1}{hw}\sum_{x=1}^{h}\sum_{y=1}^{w}{\left(L(x,y)-\mu \right)}^{2}}$$

where $\mu =\frac{1}{hw}\sum_{x,y}L(x,y)$.

Then, calculate the level of detail *D*:

The gradient is calculated using the 3 × 3 Sobel operator:

(11) $$S_{x}=L*\left[\begin{array}{ccc} -1 & 0 & 1\\ -2 & 0 & 2\\ -1 & 0 & 1 \end{array}\right],\;S_{y}=L*\left[\begin{array}{ccc} -1 & -2 & -1\\ 0 & 0 & 0\\ 1 & 2 & 1 \end{array}\right]$$

Then, the gradient amplitude is calculated:

(12) $$M(x,y)=\sqrt{S_{x}{\left(x,y\right)}^{2}+S_{y}{\left(x,y\right)}^{2}}$$

Detail Level *D*:

(13) $$D=\frac{1}{hw}\sum_{x=1}^{h}\sum_{y=1}^{w}M(x,y)$$

In CLAHE processing, CLAHE parameters are first dynamically adjusted according to local contrast *C* and detail level *D*, where *C* is used to control the threshold for contrast limitation, preventing excessive enhancement in local regions (which can amplify noise).

Contrast Limit Threshold:

(14) $$\mathrm{clipLimit}\;=\min\left(\max\left(\frac{C}{50},1.0\right),4.0\right)$$

Chunk size:

(15) $$\mathrm{tileSize}\;=\min\left(\max\left(\frac{D}{20},4\right),16\right)$$

The RGB channel is divided into sub-regions of tileSize × tileSize, and the histogram $h_{k}$ is calculated independently for each region. Truncate the histogram and reassign the pixels:

(16) $$h_{k}^{\prime }=\left\{\begin{array}{ll} h_{k} & \;\mathrm{if}\;h_{k}(i)\leq \mathrm{clipLimit}\cdot N_{\mathrm{pixel}\;}\\ \;\mathrm{clipLimit}\;\cdot N_{\mathrm{pixel}\;} & \;\mathrm{otherwise}\; \end{array}\right.\;$$

where $N_{\mathrm{pixel}\;}=\frac{hw}{{\;\mathrm{tileSize}}^{2}}$.

Then, bilinear interpolation and merging are performed, and the equalization result of each pixel is obtained as the final value through the interpolation of 4 adjacent blocks:

(17) $$I_{c}^{\mathrm{out}\;}(x,y)=\sum_{m,n=0}^{1}w_{m,n}T_{k}\left(I_{c}(x,y)\right)$$

where *k* is the adjacent block index and $w_{m,n}$ is the interpolation weight.

This method enhances contrast by a local adaptive mapping function, which has contrast sensitivity and detail adaptation characteristics, where high-contrast images (C large) reduce cropping intensity (avoid over-enhancement), and high-frequency rich regions (*D* large) use smaller blocks (preserve details).

## 3. Algorithm Verification Based on Laboratory Test

In order to deeply explore the performance of image processing algorithm in specific scenes, the effectiveness of this method is verified by controlling the extreme working conditions of water sediment concentration, lighting conditions, shooting distance, etc.

### 3.1. Experimental Image Acquisition

#### 3.1.1. Acquisition Platform

**Figure 4:**
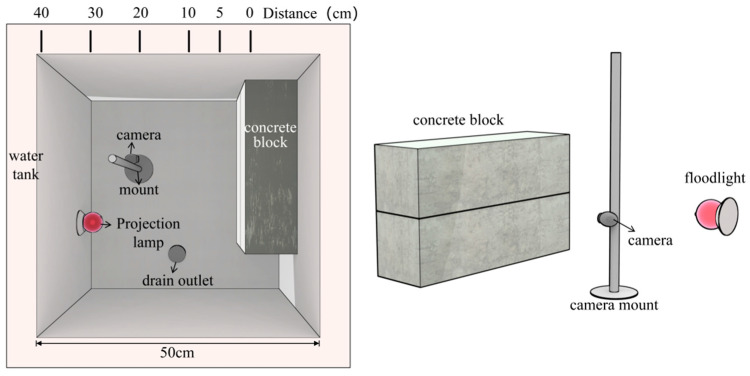
Underwater image acquisition test platform.

An image acquisition platform consisting of an underwater optical image acquisition system, an underwater dark chamber, and a cracked concrete block is designed and fabricated, as shown in Figure 4.

The platform uses an opaque water tank with an inner diameter of 50 cm × 50 cm × 60 cm (length × width × height) as a container and color-controllable spotlights as auxiliary lighting devices.

The sediment concentrations of the prepared water body are 100 g/m^3^, 200 g/m^3^, 300 g/m^3^, 400 g/m^3^ and 500 g/m^3^. Two concrete members with cracks and pockmark disease are photographed at distances of 5 cm, 10 cm, 20 cm, 30 cm, and 40 cm.

#### 3.1.2. Selection of Lighting Conditions

In related research, Chen [1] conducted comparative experiments with different light sources, and the results showed that under the same illumination conditions, compared with white light, blue light can significantly improve the average gray value, clarity, contrast, and number of key points of underwater defect images. Based on this research conclusion, the experimental design of this study selects white light and blue light as auxiliary light sources, respectively, and underwater image shooting operations are conducted at various distances.

In terms of lighting condition setting, it is specifically divided into natural lighting conditions and auxiliary light source lighting conditions:

(1) Natural lighting conditions: Set two working conditions, namely, with natural light and without natural light.

(2) Auxiliary light source lighting conditions: Programmable spotlights are selected as auxiliary lighting equipment, and white light and blue light are used for auxiliary lighting of underwater scenes, respectively.

### 3.2. Subjective Evaluation of Image Processing Effect

**Figure 5:**
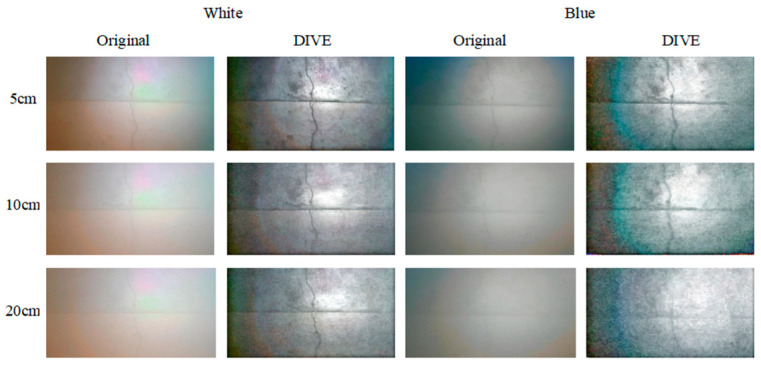
Treatment results when the sediment concentration is 500 g/m^3^ in the environment with natural light.

**Figure 6:**
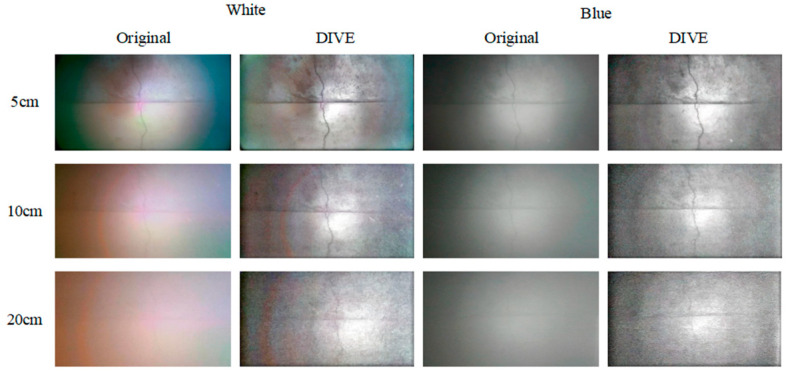
Treatment results when the sediment concentration is 500 g/m^3^ in the environment without natural light.

In this section, the photos collected under different underwater lighting conditions, water sediment concentration and shooting distance are processed by DIVE algorithm, and the effectiveness of DIVE algorithm is verified by subjective evaluation and objective evaluation indices. The pictures collected by different natural illumination, different shooting distances and different auxiliary illumination methods when the sediment concentration is 500 g/m^3^, and the processing results of DIVE algorithm are shown in Figure 5 and Figure 6.

Even in a water body with a high sediment concentration (500 g/m^3^), the DIVE algorithm can better balance the bright and dark areas of the underwater image, correct the color cast caused by the scattering of suspended particles, and improve image details and levels.

In both natural light and no natural light environments, the DIVE algorithm improves image quality most noticeably with white light and green light auxiliary illumination. In a natural light environment with a sediment content of 500 g/m^3^, the DIVE algorithm can increase the shooting distance under white light auxiliary lighting by approximately 20 cm, and the shooting distance under blue light and natural light by approximately 10 cm. In an environment without natural light, the DIVE algorithm can increase the shooting distance under white light and green light auxiliary lighting by about 10 cm. It can increase the shooting distance under red light and blue light auxiliary lighting by about 5 cm.

### 3.3. Objective Evaluation of Image Processing Effect

Due to the serious lack of information in the surrounding boundary areas of the underwater image after auxiliary illumination (dark black), this part uniformly intercepts the center area of the image (the distance from the upper, lower, left, and right boundaries is within 0.2 times the width of the original image) for quantitative evaluation.

#### 3.3.1. Objective Evaluation Indicators

Image information entropy (E) and the Underwater Color Image Quality Evaluation metric (UCIQE) [25] were adopted as objective evaluation indicators. Based on qualitative evaluation results, the impact of varying sediment concentrations on image quality was most pronounced at a shooting distance of 20 cm. Therefore, for the 10 images captured under each sediment concentration condition at this shooting distance, the mean and Standard Deviation (SD) of the objective evaluation indicators were calculated. Furthermore, the gain ratios of the objective evaluation indicators in the central region of the images processed by the DIVE algorithm were analyzed. The results are presented in Table 1.

The results show that in environments with high sediment concentrations, the algorithm demonstrates more significant improvements across all indicators. Under natural light conditions, the algorithm generally exhibits superior image quality enhancement compared to environments without natural light.

In environments lacking natural light, the gain ratios for all indicators approach 1 under sediment-free conditions, indicating that the algorithm’s processing effects are similar to the original images, with limited improvement. As sediment concentration increases, the gain ratios for each indicator gradually rise, indicating that the algorithm performs more effectively in high-sediment environments and enhances image quality. Notably, for the E (R), E (G), E, and UCIQE indicators, when sediment concentration reaches 400 g/m^3^ and 500 g/m^3^, the gain ratios exceed 1.2, indicating that the algorithm significantly enhances these indicators.

Under natural light conditions, except for a slight improvement in E (B) under sediment-free conditions, the gain ratios for the remaining indicators are close to or slightly below 1, suggesting that the processing effects are comparable to or slightly inferior to the original images. As sediment concentration increases, the gain ratios for all indicators generally rise, with greater increases observed compared to environments without natural light. The gain ratios for E (R) and E exceed 1.25 when sediment concentration is ≥300 g/m^3^, indicating that the algorithm significantly improves image quality under these conditions. The UCIQE indicator also increases with sediment concentration, though the overall improvement is slightly lower than that for E (R) and E. The E (G) and E (B) indicators similarly show an upward trend, but the magnitude of improvement is relatively smaller.

#### 3.3.2. Number of Key Points

Under varying sediment concentration conditions, the number of SIFT keypoints extracted from 10 images captured at a shooting distance of 20 cm was statistically analyzed to evaluate the enhancement effects of each processing stage on image features. The changes in the mean and standard deviation of the keypoint count in the central region of the images across different processing stages are presented in Table 2.

Analyzing the above chart yields the following findings:

(1) In environments without natural light and under sediment-free conditions, the number of keypoints significantly increased from the original images to those after detail enhancement, indicating that the processing pipeline effectively enhanced image features. As sediment concentration increased, the number of keypoints in the original images dropped sharply, but after processing (especially during the detail enhancement stage), the keypoint count still rebounded significantly, demonstrating that the algorithm could effectively extract image features even in high-sediment environments. The detail enhancement step had the most pronounced effect on increasing the keypoint count, highlighting its importance in environments without natural light.

(2) In natural light environments and under sediment-free conditions, the number of keypoints gradually increased throughout the processing stages, though the increase was relatively small, suggesting that the processing provided some assistance in feature extraction but was less effective than in environments without natural light. As sediment concentration rose, the number of keypoints in the original images also decreased, but after processing, it remained higher than in the original images, indicating that the algorithm could still improve feature extraction capabilities in natural light environments. Detail enhancement remained the step with the most significant improvement, though the overall increase was lower than in environments without natural light.

(3) The gain ratio in the number of keypoints was generally lower in natural light environments compared to those without natural light, as the original images in natural light already had a higher number of keypoints. As sediment concentration increased, the gain ratio still showed an upward trend, but the increase was relatively small.

(4) Each processing step significantly increased the number of keypoints. Color correction, which only involved minor adjustments to the red and green channels, had a relatively smaller impact on increasing the keypoint count.

### 3.4. Modular Ablation Analysis and Feature Extraction Verification

**Figure 7:**
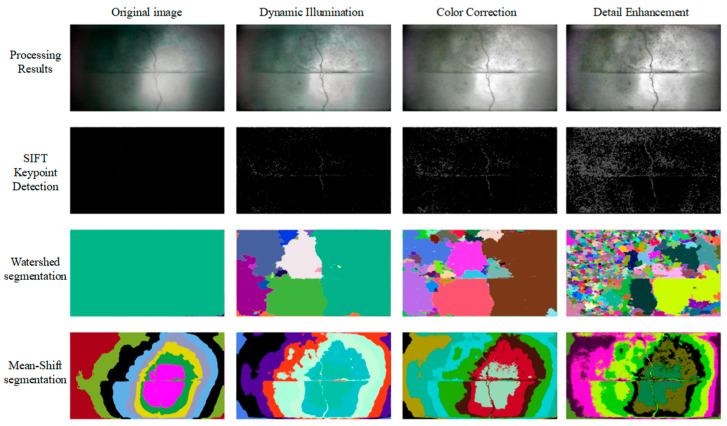
Variation in image features with processing.

**Figure 8:**
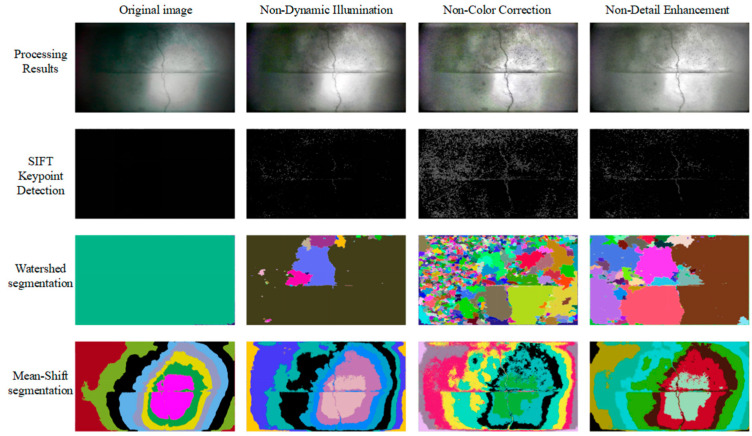
Image features without a certain step.

Two basic image segmentation methods, SIFT key point detection, Watershed segmentation, and Mean-Shift segmentation, are used to extract image features step by step. The results are shown in Figure 7. After eliminating each step, feature extraction is performed on the processing result, and the results are shown in Figure 8.

As can be seen from Figure 7, the dynamic lighting module basically makes the key features of cracks in the image appear, and the key points and image segmentation boundaries can well reflect the key information in the image, and also present some pockmarks (small potholes) on the concrete surface. The color correction part of the visual enhancement module only adds more detailed features to the edge area of the image, and its main function is to balance the color distribution, but it has no obvious effect on the enhancement of feature information in the image. The detail enhancement part in the visual enhancement module further enhances image details based on pre-processing, primarily improving the information content of small holes, pocket surfaces, and other features on the concrete surface.

It can be seen from Figure 8 that after the dynamic illumination module is eliminated, the crack features in the image are difficult to mine through color correction and detail enhancement due to the smooth transition of illumination and cracks. However, after eliminating the color correction part in the visual enhancement module, the processing results of the image are basically the same as those before elimination. After eliminating the detail enhancement part in the visual enhancement module, it is difficult to dig small holes and pockmarks on the concrete surface.

In addition, a large amount of noise appears in over-dark areas in Figure 7 and Figure 8, which are mainly caused by local detail enhancement operations. When enhancing the image, in order to highlight key features such as cracks, a local detail enhancement algorithm is adopted. However, the dark areas in the original image itself hardly contain valid information. After enhancement, the algorithm will try to dig out “details” from these areas with almost no information, resulting in a lot of noise, which may be mistakenly identified as key points, such as noise such as sand.

Comprehensive analysis shows that each processing module of the DIVE algorithm plays an irreplaceable key role in the process of image enhancement:

The dynamic illumination module serves as the basis for feature extraction. By eliminating the influence of underwater non-uniform lighting, key structural features such as cracks are highlighted from the background, providing clear initial images for subsequent processing; Although the direct effect of the color correction module on feature enhancement is limited, it significantly improves the detail recognizability of the edge area of the image by balancing the color distribution, and provides more accurate color information for subsequent analysis. As the final optimization link, the detail enhancement module specifically strengthens the small defects (such as holes and pockets) on the concrete surface, which greatly improves the amount of information and detection accuracy of the image.

The absence of the dynamic illumination module will cause the overall features of the image to be blurred. In contrast, the local optimization function of the visual enhancement module further compensates for the deficiency in lighting processing. This stepped processing architecture (global lighting correction → color balance → local detail enhancement) ensures the robustness of the algorithm in different underwater environments and provides complete technical support for the accurate detection of surface defects of concrete structures.

### 3.5. Comparison of Underwater Image Enhancement Algorithms

**Figure 9:**
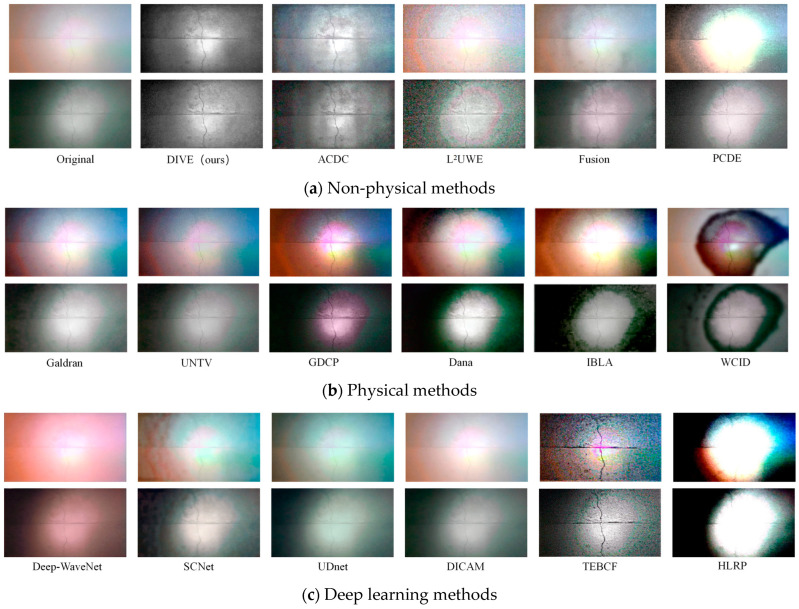
Comparison of underwater image enhancement algorithms.

According to the classification of underwater image enhancement methods, non-physical methods, physical methods, and deep learning methods were employed to process two images—one captured under natural light with white light auxiliary illumination and the other under non-natural light with blue light auxiliary illumination, both at a sediment concentration of 400 g/m^3^ and a shooting distance of 10 cm. The results are shown in Figure 9.

For non-physical methods, four approaches—ACDC [15], L^2^UWE [26], Fusion [27], and PCDE [17]—were compared. For physical methods, six approaches—Galdran [28], UNTV [29], GDCP [16], Dana [30], IBLA [31], and WCID [32]—were evaluated. For deep learning methods, six models—Deep-WaveNet [21], SCNet [33], UDnet [22], DICAM [34], TEBCF [35], and HLRP [36]—were contrasted.

Among non-physical methods, the image processed by DIVE (ours) demonstrated notable improvements in detail and clarity, with natural color restoration that effectively reduced blur and color cast in underwater images. ACDC improved overall image brightness but left some details blurry, with mediocre color restoration. L^2^UWE performed well in enhancing brightness and contrast but suffered from over-enhancement, leading to color distortion in some areas and introducing significant noise. Fusion integrated multiple types of information, achieving good detail and color restoration but introducing some noise, which reduced contrast and detail. PCDE exhibited abnormal contrast adjustment, effectively highlighting objects in the image but requiring improvement in color restoration.

Among physical methods, Galdran-processed images displayed vibrant colors and improved contrast but performed poorly in detail restoration. UNTV effectively restored image details with natural colors but suffered from local overexposure or underexposure under uneven lighting conditions. GDCP, Dana, IBLA, and WCID all showed some effectiveness in color restoration and detail enhancement but exhibited noticeable abnormalities in brightness processing, with dark region information almost entirely lost.

Most complex deep learning algorithms were trained and optimized using images from shallow marine waters, and this study directly applied their pre-trained models for processing. Consequently, these models performed poorly on dark, turbid water images. Deep-WaveNet exhibited obvious color distortion due to its over-enhancement of the red channel, as marine waters typically appear light blue or green, and it failed to address uneven illumination. SCNet predicted and compensated for dark regions in the image but introduced multiple abnormal halos. UDnet demonstrated abnormal color correction and had minimal effect on detail enhancement or uneven illumination. DICAM showed no significant changes. TEBCF’s processing results closely resembled those of L^2^UWE in non-physical methods, with excessive detail enhancement introducing substantial noise. HLRP, incorporating reflection priors (i.e., physical information) into its model, produced results similar to those of GDCP, Dana, IBLA, and WCID in physical methods, with abnormal illumination estimation exacerbating the imbalance between dark and bright regions after processing.

## 4. Engineering Application Research

Images acquired at the engineering site and representative images from the publicly available dataset are used for processing and evaluation to verify the practical application effect of the DIVE method.

### 4.1. Acquisition of Images on Site

**Figure 10:**
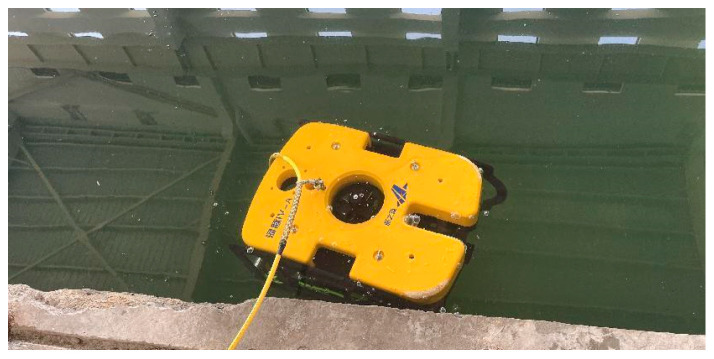
Underwater image detection site.

**Figure 11:**
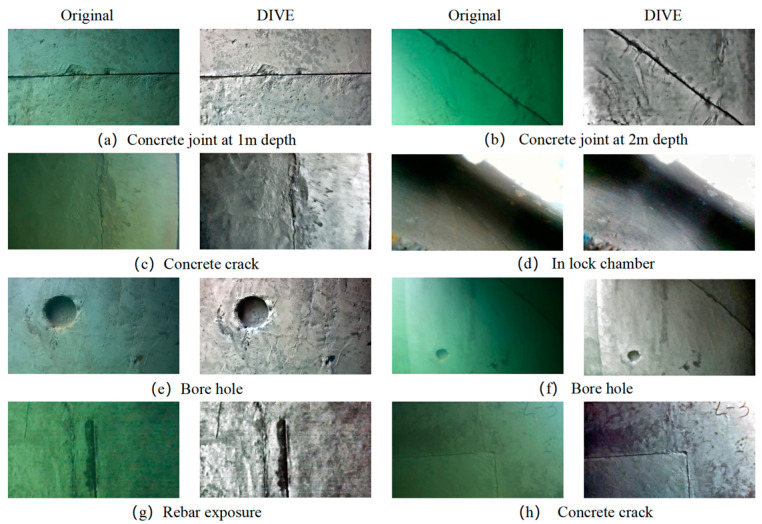
Original image and DIVE processing results of the concrete side wall of a sluice sedimentation tank.

An underwater robot is used to collect images of the concrete side wall of a sluice sedimentation tank, and the collection site is shown in Figure 10. Using the DIVE algorithm proposed in this paper for processing, part of the original images and the processing results obtained are shown in Figure 11.

The DIVE algorithm can effectively handle the color deviation of underwater images, restore the primary colors of concrete structures, and simultaneously improve the feature extraction range of underwater images while reducing the blurring degree of the images. In addition, the dynamic illumination module proposed in this paper can significantly improve the information loss caused by uneven illumination and improve the overall information amount of the image, as shown in Figure 11c,f,h.

### 4.2. Marine Image Dataset Images

**Figure 12:**
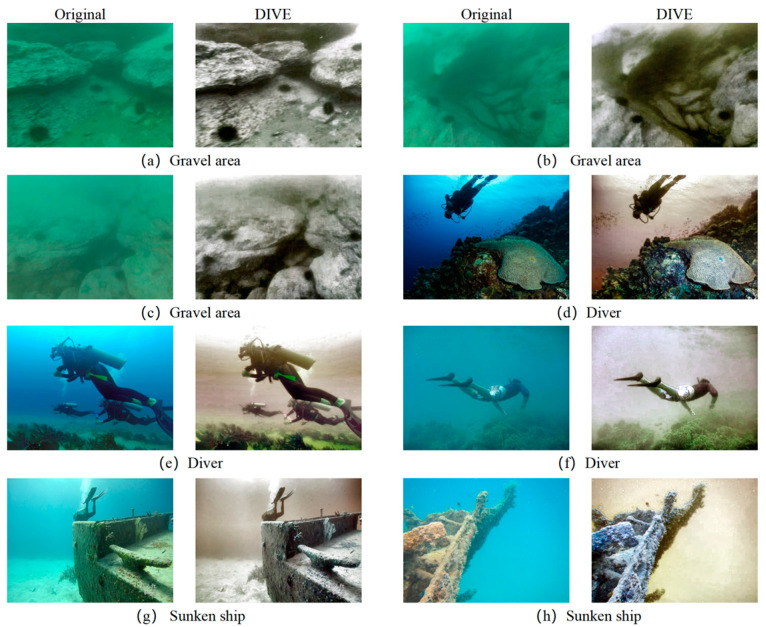
Images and processing results of the ocean image dataset.

Images with uneven illumination, color cast, and turbidity characteristics are selected from Realworld-Underwater-Image-Enhancement (RUIE) [37] and UFO-120 [38] for processing. Part of the original pictures and processing results are shown in Figure 12.

Compared with images collected in laboratory experiments and sedimentation tanks, the ocean images with an obvious color shift in the dataset clearly reflect the effect of dynamic decision-making on the G/B channel mean in DIVE, indicating that color correction is still necessary.

At the same time, the DIVE algorithm has a very good processing effect for the over-dark area in the seabed image caused by the auxiliary illumination, as shown in Figure 11d.

Comprehensive analysis of the processing results of laboratory test images, sedimentation tank images, and ocean image dataset images shows that the illumination–scattering decoupling processing framework of the DIVE method has better flexibility and generalization, and can significantly improve the underwater image quality under different working conditions.

## 5. Discussion

The DIVE algorithm innovatively constructs innovations in physical mechanisms, algorithm architecture, and engineering implementation through the illumination–scattering decoupling processing framework, effectively solving the triple contradiction of existing underwater image enhancement methods. It has significant advantages when processing surface defect images of underwater concrete structures.

(1) Concrete surface images under different shooting distances, sediment concentrations, and lighting conditions were collected through laboratory experiments to simulate the concrete surface images of reservoir dams with high sediment content and great water depth.

(2) The decoupled design of the DIVE dynamic illumination module (for brightness correction) and the visual enhancement module (for color/detail restoration) overcomes the limitations of traditional methods, such as halo artifacts in Retinex and dark channel failure in DCP.

(3) Through downsampling, separation convolution, and parallel computing, about 25FPS processing is realized on embedded devices to meet the real-time inspection needs of underwater robots.

(4) Through laboratory tests, engineering sites (sluice sedimentation tanks) and public datasets (RUIE, UFO-120) images in different underwater environments and various evaluation index verification, the DIVE algorithm can balance the bright areas and dark areas of underwater images, correct color cast, improve image details and levels, and perform well in qualitative evaluation, quantitative evaluation (objective evaluation indicators and number of key points) and feature extraction verification. The underwater image processing results under different working conditions verify the robustness of the algorithm, especially for the detection of concrete cracks, holes, and other defects.

It provides an efficient and reliable image enhancement tool for intelligent inspection of underwater structures, and can be extended to marine engineering, dam monitoring and other fields.

## Figures and Tables

**Table 1:** Mean and Standard Deviation of Gain Ratios for Objective Evaluation Indicators in the Central Region of Images.

Category	Sediment Concentration	E (R)		E (G)		E (B)		E		UCIQE	
		Mean	SD	Mean	SD	Mean	SD	Mean	SD	Mean	SD
No natural light environment	0	1.19	0.0025	1.13	0.0032	1.11	0.0028	1.16	0.0036	1.09	0.0021
	100 g/m^3^	1.11	0.0036	1.2	0.0028	1.16	0.0031	1.12	0.0039	1.03	0.0038
	200 g/m^3^	1.17	0.0028	1.13	0.0033	1.12	0.0029	1.17	0.0032	1.14	0.0025
	300 g/m^3^	1.18	0.0026	1.25	0.0021	1.2	0.0024	1.28	0.002	1.1	0.0032
	400 g/m^3^	1.24	0.0021	1.3	0.0018	1.29	0.0019	1.22	0.0028	1.21	0.0023
	500 g/m^3^	1.27	0.0019	1.19	0.0029	1.2	0.0026	1.22	0.0027	1.18	0.0026
Natural light environment	0	1.16	0.0031	1.14	0.0034	1.17	0.0029	1.06	0.0042	1.02	0.0041
	100 g/m^3^	1.27	0.002	1.24	0.0023	1.21	0.0025	1.17	0.0031	1.17	0.0028
	200 g/m^3^	1.22	0.0024	1.19	0.0027	1.18	0.0028	1.14	0.0034	1.15	0.003
	300 g/m^3^	1.27	0.0018	1.27	0.0019	1.22	0.0023	1.29	0.0018	1.22	0.0022
	400 g/m^3^	1.27	0.0019	1.3	0.0017	1.27	0.002	1.24	0.0025	1.17	0.0027
	500 g/m^3^	1.3	0.0016	1.28	0.0018	1.24	0.0021	1.27	0.002	1.13	0.0031

**Table 2:** Changes in the Mean and Standard Deviation of Keypoint Count in the Central Region of Images Across Processing Stages.

Category	Sediment Concentration	Original Image		Dynamic Illumination		Color Correction		Detail Enhancement	
		Mean	SD	Mean	SD	Mean	SD	Mean	SD
No natural light environment	0	153.04	12.67	285.42	10.89	533.49	14.22	1116.89	61.36
	100 g/m^3^	66.62	5.56	140.69	4.36	266.78	8.23	639.46	30.02
	200 g/m^3^	10.47	0.38	60.23	2.89	186.75	9.78	716.31	8.02
	300 g/m^3^	8.85	0.36	70.42	3.78	243.68	10.89	738.45	10.03
	400 g/m^3^	1.14	0.05	49.38	3.47	229.22	10.23	718.07	6.35
	500 g/m^3^	0.67	0.04	28.92	3.65	255.96	9.33	661.24	4.36
Natural light environment	0	367.39	23.11	475.62	14.23	871.68	21.33	1328.20	123.36
	100 g/m^3^	33.74	4.36	221.33	8.22	556.86	12.45	924.84	43.36
	200 g/m^3^	7.42	0.31	99.93	5.56	370.89	10.45	739.30	14.70
	300 g/m^3^	1.21	0.05	123.43	7.34	512.40	11.78	876.70	25.35
	400 g/m^3^	1.03	0.04	66.71	5.11	343.33	10.00	658.92	12.02
	500 g/m^3^	0.23	0.01	75.72	6.00	402.79	4.22	855.90	16.69

## Data Availability

The datasets supporting this study are available from the corresponding author on reasonable request.

## Abbreviations

The following abbreviations are used in this manuscript:

DIVE	Dynamic Illumination and Vision Enhancement
ROV	Remote Operated Vehicle
DCP	Dark Channel Prior
CLAHE	Contrast Limited Adaptive Histogram Equalization
ACDC	Attenuated Color Channel Correction and Detail Preserved Contrast Enhancement
RUIE	Realworld-Underwater-Image-Enhancement
SD	Standard Deviation

## References

[1] Chen D., Kang F., Chen J., Zhu S., Li H. (2024). Effect of light source wavelength on surface defect imaging in deep-water concrete dams. NDT E Int..

[2] Hu J., Wang C., Ma F. (2022). Comprehensive analysis for leakage cause of the entry and outlet sections of the water-conveyance crossing structures. Hydro-Sci. Eng..

[3] Verma G., Kumar M. (2022). Systematic review and analysis on underwater image enhancement methods, datasets, and evaluation metrics. J. Electron. Imaging.

[4] Aguirre-Castro O., García-Guerrero E., López-Bonilla O., Tlelo-Cuautle E., López-Mancilla D., Cárdenas-Valdez J., Olguín-Tiznado J., Inzunza-González E. (2022). Evaluation of underwater image enhancement algorithms based on Retinex and its implementation on embedded systems. Neurocomputing.

[5] Ramalingam S.P., Kumar V. (2023). Automatizing the generation of building usage maps from geotagged street view images using deep learning. Build. Environ..

[6] Xiao S., Shen X., Zhang Z., Wen J., Xi M., Yang J. (2024). Underwater image classification based on image enhancement and information quality evaluation. Displays.

[7] Espinosa A.R., McIntosh D., Albu A.B. An efficient approach for underwater image improvement: Deblurring, dehazing, and color correction. Proceedings of the IEEE/CVF Winter Conference on Applications of Computer Vision.

[8] Han F., Qiu X., Zhao W., Xue Y., Yuan L., Peng X., Zhao Y., Zhang J. (2024). A solution for the automatic detection of expansion joints in dam stilling pools using underwater robots. Eng. Struct..

[9] Li F., Wan L., Zheng J., Wang L., Xi Y. (2025). Contrastive Feature Disentanglement via Physical Priors for Underwater Image Enhancement. Remote. Sens..

[10] Liu J., Liu Y., Jiang Q. (2025). Delving into Underwater Image Utility: Benchmark Dataset and Prediction Model. Remote Sens..

[11] Ning Y., Jin Y.P., Peng Y.D., Yan J. (2023). Low-illumination underwater image enhancement based on non-uniform illumination correction and adaptive artifact elimination. Front. Mar. Sci..

[12] Sachin S.S., Sunil D.S. (2016). Nonuniform Illumination Correction Algorithm for Underwater Images Using Maximum Likelihood Estimation Method. J. Eng..

[13] Anwar S., Li C., Porikli F. (2018). Deep Underwater Image Enhancement. arXiv.

[14] Fu X., Zhuang P., Huang Y., Liao Y., Zhang X.-P., Ding X. A retinex-based enhancing approach for single underwater image. Proceedings of the 2014 IEEE International Conference on Image Processing (ICIP).

[15] Zhang W., Wang Y., Li C. (2022). Underwater Image Enhancement by Attenuated Color Channel Correction and Detail Preserved Contrast Enhancement. IEEE J. Oceanic Eng..

[16] Peng Y.-T., Cao K., Cosman P.C. (2018). Generalization of the Dark Channel Prior for Single Image Restoration. IEEE Trans. on Image Process..

[17] Wang S., Ma K., Yeganeh H., Wang Z., Lin W. (2015). A Patch-Structure Representation Method for Quality Assessment of Contrast Changed Images. IEEE Signal Process. Lett..

[18] He K., Sun J., Tang X. (2011). Single Image Haze Removal Using Dark Channel Prior. IEEE Trans. Pattern Anal. Mach. Intell..

[19] Liu S., Fan H., Lin S., Wang Q., Ding N., Tang Y. (2022). Adaptive Learning Attention Network for Underwater Image Enhance. IEEE Robot. Autom. Lett..

[20] Naik A., Swarnakar A., Mittal K. (2021). Shallow-UWnet: Compressed Model for Underwater Image Enhancement. Proc. AAAI Conf. Artif. Intell..

[21] Sharma P., Bisht I., Sur A. (2023). Wavelength-based attributed deep neural network for underwater image restoration. ACM Trans. Multimed. Comput. Commun. Appl..

[22] Saleh A., Sheaves M., Jerry D., Azghadi M.R. (2025). Adaptive Deep Learning Framework for Robust Unsupervised Underwater Image Enhancement. Expert Syst. Appl..

[23] Zhao M., Hu C., Wei F., Wang K., Wang C., Jiang Y. (2019). Real-Time Underwater Image Recognition with FPGA Embedded System for Convolutional Neural Network. Sensors.

[24] Yang H., Xu J., Lin Z., He J. (2024). LU2Net: A Lightweight Network for Real-time Underwater Image Enhancement. arXiv.

[25] Yang M., Sowmya A. (2015). An Underwater Color Image Quality Evaluation Metric. IEEE Trans. Image Process. A Publ. IEEE Signal Process. Soc..

[26] Marques T.P., Branzan Albu A. (2020). L2 UWE: A Framework for the Efficient Enhancement of Low-Light Underwater Images Using Local Contrast and Multi-Scale Fusion. Proceedings of the 2020 IEEE/CVF Conference on Computer Vision and Pattern Recognition Workshops (CVPRW).

[27] Ancuti C., Ancuti C.O., Haber T., Bekaert P. (2012). Enhancing Underwater Images and Videos by Fusion. Proceedings of the 2012 IEEE Conference on Computer Vision and Pattern Recognition.

[28] Galdran A., Pardo D., Picón A., Alvarez-Gila A. (2015). Automatic Red-Channel underwater image restoration. J. Vis. Commun. Image Present..

[29] Xie J., Hou G., Wang G., Pan Z. (2022). A Variational Framework for Underwater Image Dehazing and Deblurring. IEEE Trans. Circuits Syst. Video Technol..

[30] Berman D., Treibitz T., Avidan S. (2017). Diving into haze-lines: Color restoration of underwater images. Proc. Br. Mach. Vis. Conf. (BMVC).

[31] Peng Y.-T., Cosman P.C. (2017). Underwater Image Restoration Based on Image Blurriness and Light Absorption. IEEE Trans. Image Process.

[32] Chiang J.Y., Chen Y.C. (2012). Underwater Image Enhancement by Wavelength Compensation and Dehazing. IEEE Trans. Image Process..

[33] Fu Z., Lin X., Wang W., Huang Y., Ding X. Underwater image enhancement via learning water type desensitized representations. Proceedings of the ICASSP 2022-2022 IEEE International Conference on Acoustics, Speech and Signal Processing (ICASSP).

[34] Tolie H.F., Ren J., Elyan E. (2024). DICAM: Deep Inception and Channel-Wise Attention Modules for Underwater Image Enhancement. Neurocomputing.

[35] Yuan J., Cai Z., Cao W. (2022). TEBCF: Real-World Underwater Image Texture Enhancement Model Based on Blurriness and Color Fusion. IEEE Trans. Geosci. Remote Sens..

[36] Zhuang P., Wu J., Porikli F., Li C. (2022). Underwater Image Enhancement With Hyper-Laplacian Reflection Priors. IEEE Trans. Image Process.

[37] Liu R., Fan X., Zhu M., Hou M., Luo Z. (2020). Real-World Underwater Enhancement: Challenges, Benchmarks, and Solutions Under Natural Light. IEEE Trans. Circuits Syst. Video Technol..

[38] Islam M.J., Luo P., Sattar J. (2020). Simultaneous Enhancement and Super-Resolution of Underwater Imagery for Improved Visual Perception. arXiv.

